# Transgenic plant generated by RNAi-mediated knocking down of soybean *Vma12* and *soybean mosaic virus* resistance evaluation

**DOI:** 10.1186/s13568-020-00997-6

**Published:** 2020-04-06

**Authors:** Hexiang Luan, Wenlin Liao, Yingpei Song, Haopeng Niu, Ting Hu, Haijian Zhi

**Affiliations:** 1 National Center for Soybean Improvement, Nanjing Agricultural University, Nanjing, 210095 Jiangsu China; 2 College of Life Sciences, Institute of Plant Genetic Engineering, Qingdao Agricultural University, Qingdao, 266109 China; 3 School of Pharmacy and Life Sciences, Jiujiang University, Jiujiang, 332000 Jiangxi China

**Keywords:** Soybean, Soybean mosaic virus, P3, RANi, Vma12

## Abstract

Soybean mosaic virus (SMV) is one of the most destructive viral diseases in soybean and causes severe reduction of soybean yield and destroys the seed quality. However, the production of SMV resistant plants by transgenic is the most effective and economical means. Based on our previous yeast two-hybrid assay, the *GmVma12* was selected as a strong candidate gene for further function characterization. Here we transformed soybean plants with a construct containing inverted repeat of-*GmVma12* sequence to analyze the role of *GmVma12* during SMV invasion. Totals of 33 T_0_ and 160 T_1_ plants were confirmed as positive transgenic plants through herbicide application, PCR detection and LibertyLink^®^ strip screening. Based on the segregation ratio and Southern Blot data, T_1_ lines No. 3 and No. 7 were selected to generate T_2_ plants. After SMV-SC15 inoculation, 41 T_1_ and 38 T_2_ plants were identified as highly resistant, and their quantification disease levels were much lower than non-transformed plants. The transcript level of *GmVma12* in T_2_ plants decreased to 70% of non-transformed plants. The expression level of SMV-*CP* transcript in T_2_ transgenic plants was lower than that in non-transformed plants and SMV CP protein in T_2_ plants could not be detected by Enzyme-linked Immunosorbent assay, which indicated that SMV production would be inhibited in transgenic plants. Moreover, coat mottles of T_2_ seeds were obliterated significantly. In conclusion, inverted repeat of the hairpin structure of *GmVma12* interfered with the transcription of *GmVma12*, which can induce resistance to SMV in soybean. This research lays the foundation for the mechanism of SMV pathogenesis, and provides new ideas for SMV prevention and control.

## Introduction

Soybean (*Glycine max* (L.) Merr.) originated from China has been cultivated for more than five thousand years, supplying plant fat and protein in human daily diet. *Soybean mosaic virus* (SMV) infection is prevalent from north spring soybean production area to the Yangtze River valley area in China (Li et al. 2010). Once SMV infects soybean, leaves are damaged severely, which leads to the reduction of photosynthetic production, nutrient absorption and transport capacity. Ultimately SMV affects the grain traits, resulting in 10–30% yields reduction, or even crop failure (Liao et al. 2002). One potential viable approach to prevent SMV infections in soybeans is by identifying resistance genes and breeding SMV resistant soybean cultivars.

*Agrobacterium* is a naturally occurring microorganism that can serve as transgenic vector in nature (Hadi et al. 1996). *Agrobacterium*-mediated transformation, due to many advantages such as low cost, low gene copy number and genetic stability, has become a popular method in crop breeding (Hansen et al. 1999). It was reported that the first transgenic soybean was produced in 1988 using Agrobacterium-mediated transformation and since then, this technology has been widely used in soybean transgenic research (Hinchee et al. 1988). Currently, using *agrobacterium* transformation to induce RNAi-mediated resistance to SMV is a popular strategy in China. The RNAi strategy was used to trigger robust resistance against three viruses in soybean plants by expressing several short inverted repeat of a portion of the virus sequences (Zhang et al. 2011). The construct containing inverted repeat of SMV-*HC*-*Pro* was transformed into five soybean genotypes. The transgenic soybeans demonstrated strong resistant to SMV in transgenic plants (Gao et al. 2015). In addition, the transgenic lines with silenced P3 cistron by RNAi showed significantly increased resistance to multiple potyvirus strains and isolates (Yang et al. 2018).

Resistance genes can be divided into dominant resistance genes and recessive resistance genes according to genetic criteria (Robaglia et al. 2006). Viral infection can be inhibited when either the dominant resistance gene or the recessive resistance gene in the host changes (Fraser et al. 1985). The host dominant resistance (R) gene, which codes a protein that recognizes virus avirulence protein (Avr), can trigger hypersensitive response upon virus infection and/or extreme resistance (ER) either through direct or indirect interactions with Avr, which can lead to, systemic resistance for the host against the virus (Bonas et al. 2002; Goldbach et al. 2003; Soosaar et al. 2005). The host recessive resistance genes can prevent the virus infection by altering a single or a few amino acids of the encoded protein, causing functional changes in the host target that are essential for virus survival, which inhibits the replication and invasion of virus (Fraser 1990; Johansen et al. 2001). Most of the known dominant R genes play a role in resistance to fungi and bacteria (Wang et al. 2012), whereas recessive resistance genes are generally found in virus studies (Truniger et al. 2009). Our previous study found from the yeast two-hybrid assay that a soybean transmembrane protein 199 (GmVma12), a factor of vacuolar-ATPase (V-ATPase), interacted with the effector P3 protein of SMV and participated in the reproduction of SMV (Luan, et al. 2019). It was speculated that *GmVma12* acted as recessive resistance gene and was essential to the resistance of soybean to SMV.

In order to reveal the role of *GmVma12* during SMV invasion, two isoforms of *GmVma12* were compared and the conservative sequence was determined, which was chosen to construct the RNAi expression vector pB7GWIWG2(II)-*GmVma12i*. Then, transgenic soybean seedlings were obtained via *agrobacterium*-mediated soybean cotyledonary-node transformation. The resistance of transgenic plants to SMV was evaluated by investigating phenotypes of T_1_ and T_2_ plants and by PCR, southern blot, and protein detection. This study demonstrated that interfering the transcription of *GmVma12* could enhance the resistance to SMV in soybean plant.

## Materials and methods

### Plant growth and virus strains

The soybean cultivar Tianlong No.1 was used for *agrobacterium*-mediated soybean cotyledonary node transformation. The seeds were surface-sterilized by chlorine gas in a tightly sealed vacuum dryer for 8 h. The soybean cultivar Williams 82 was used to amplify *GmVma12* RNAi segment. SMV strain SC15 provided by the National Center for Soybean Improvement (NCSI, Nanjing Agricultural University, Nanjing, China) was used for infecting transgenic soybean seedlings.

### RNAi segment cloning and vector construction

The coding sequences of two *GmVma12* isoforms were obtained by 5′ RACE from yeast two hybrid contigs and aligned by Lasergene software to determine the consensus sequence. The forward (5′-GGGGACAAGTTTGTACAAAAAAGCAGGCTTCGGTCGGGTTAGTGATATCCA-3′) and reverse primers (5′-GGGGACCACTTTGTACAAGAAAGCTGGGTCAGTCGGGTCAAGTCGGGTCT-3′) primers were designed to amplify the 189-bp fragment *GmVma12* consensus sequence with an attB adaptor from Williams 82 cDNA. The PCR products were inserted into the entry vector pDONR™221 (Invitrogen, USA), and cloned into destination hairpin vector pB7GWIWG2(II) (Karimi et al. 2002) (Additional file 1: Fig. S1) according to the GATEWAY™ protocol. All of the constructions after BP and LR reaction were sequenced to guarantee the correct ligation. The constructed vector was transformed into *A. tumefaciens* strain EHA105 using electric shock methods (Höfgen et al. 1988) for soybean transformation.

### Soybean transformation

The cotyledonary node-Agrobacterium-mediated soybean transformation system used in this study was described by Gao (Gao et al. 2015). Briefly, the transformed EHA105 colony was inoculated into 200 mL YEB liquid medium, and cultured at a speed of 200 rpm/min at 28 °C until OD_600nm_ was up to 1.0–1.2. Four 50 ml Falcon tubes were used to centrifuge bacteria culture at a speed of 4000 rpm for 10 min, and the supernatant was discarded. Cell pellet was resuspended in suitable liquid co-culture medium (LCCM), and the OD_600nm_ value was adjusted to 0.6–0.8.

Twenty sterilized seeds were inserted onto the germination medium (GM). After incubation for 15 h, each seed was subjected to coat removal, after which they were split evenly into two explants containing the cotyledons and hypocotyls by a longitudinal cut along the hilum. The explants were immersed in the prepared *A. tumefaciens* LCCM for 30 min at room temperature, transferred to CCM and incubated at 23–25 °C without light for 5 d. The elongated hypocotyl was cut to 5–7 mm in length and elongated on shoot induction medium (SIM) to grow for 4 weeks, which was changed to new SIM in the third week. The thriving explants were embedded in shoot elongation medium (SEM) and subcultured per 2 weeks for 5–6 times until the shoot had elongated to 3–5 mm. The green resistant shoots were then transferred to rooting medium (RM). After 2–3 weeks, the strong shoots were washed out from bottles individually and transplanted to plastic pots with nutrient soil and vermiculite and then wrapped by plastic membrane with several holes for acclimatization in a growth chamber at 25 °C with an 18 h photoperiod for 1–2 weeks. Robust seedlings were moved to a greenhouse and prepared for further analyses. When the seedlings had grown up to the top, the plastic membrane was removed and regenerated seedlings with developed roots were transferred to greenhouse for further growth. The recipes of mediums were described in Additional file 1: Table S1.

### Screening positive transgenic seedlings

The transgenic seedlings were identified by polymerase chain reaction (PCR) to detect the existence of transgene fragments, leaf-painting assay to test the resistance to herbicide and LibertyLink^®^ strip (QuickStix™ Kit purchased from EnviroLogix Inc., cat #AS 013 LS, Portland, ME, USA) to check the expression of *bar* gene. The seedlings confirmed to be positive by three methods were identified as positive transgenic plants.

The primers were designed by primer premier 5.0 for *GmVma12* interference segment (*GmVma12i*), 35S promoter (F: 5′-GCTCAACACATGAGCGAAAC-3′, R: 5′-GACGCACAATCCCACTATCC-3′) and *bar* (F: 5′-CGAGACAAGCACGGTCAACTT-3′, R: 5′-AAACCCACGTCATGCCAGTTC-3′). The DNA of transgenic seedlings was used as templates for PCR reaction. Twenty microliter PCR reaction system contained 1 μL sample DNA, 1 μL forward primer, 1 μL reverse primer, 12.5 μL 2 × Taq Master Mix and 9.5 μL ddH_2_O. The PCR program was as following: 94 °C for 2 min, 35 circles of 98 °C for 10 s, 58 °C for 20 s, 72 °C for 30 s and a final extension at 72 °C for 2 min. The estimated lengths of fragments were 250-bp (*GmVma12i*), 486-bp (35S) and 414-bp (*bar*). The half-leaves were painted with diluted herbicide phosphinothricin (PPT) at 200 mg/L with 0.01% Tween-20 and left the other half as untreated control. The symptom of herbicide was observed in 3–5 d. The 1 cm^2^ leaf tissue was collected into 1.5 mL centrifugation tube and ground-up fully by pestle rotation, 0.5 mL extraction buffer was added into tube and one LibertyLink^®^ strip was inserted into the tube. The strips with two lines (control line and test line) were considered to be positive, while those with only control line were negative. Southern blot hybridizations were conducted for further confirmation (see below).

### Southern blot analysis

The total genomic DNA of T_1_ plants (screened according to resistance to SMV and PPT) was isolated by cetyltrimethylammonium bromide (CTAB) method and digested by Hind III (Thermo, Waltham, MA, USA). The digestion product were separated via 0.8% agarose gel electrophoresis and blotted in Hybond-N ^+^ nylon membrane (Amersham, Buckinghamshire, UK). The primers F: 5′-GAGAATTAAGGGAGTCACGTTATG-3′ and R: 5′-CGTTGCGTGCCTTCCAG-3′ were used to amplify 538-bp *bar* from pB7GWIWG2(II). The segment was tag labeled with DIG-high prime (Roche, USA) as probe. The prehybridization, hybridization, washing, detection and exposure were operated according to the manual of DIG High Prime DNA Labeling and Detection Starter Kit II (Roche, Indianapolis, IN, USA).

### Virus inoculation and resistance evaluation

Several fresh SMV-SC15 leaves were picked and crashed in sterilized vessel with 20 mL PBS buffer. The true leaves of transgenic seedlings were inoculated with virus buffer by a hairbrush which was used to rub the foliage softly. The leaves were moisturized after inoculation to revitalize the seedlings. The resistance to SC15 of T_1_ and T_2_ transgenic seedlings was observed on first (V_1_), second (V_2_), third (V_3_) and fourth (V_4_) trifoliate within 3 weeks. The resistance was classified into high resistance (HR), delayed resistance (DR), mild resistance (MR) and susceptible (S) according to the macroscopic symptoms. When the seedlings were in reproductive growth period, the symptoms of top three trifoliate were also investigated and classified into 5 ratings (Fig. 4c).

### Quantitative real-time PCR (qRT-PCR) analysis

The RNA of T_2_ transgenic seedlings at 7 days and 14 days after infecting SMV was isolated by Takara RNA Extraction kit and reverse transcript was synthesized by PrimeScript^®^ kit. The qRT-PCR primers of *GmVma12*, *CP* and *GmeTubulin* were shown in Additional file 1: Table S2. The reactions were carried out with SYBR^®^ Premix Ex Taq™ kit on Roche LC 480II qRT-PCR machine. The reaction mixture was composed of 2 μL sample DNA, 0.4 μL forward primer, 0.4 μL reverse primer, 10 μL 2 × SYBR^®^ PremixEx *Taq* and 7.2 dd H_2_O. The reactions were performed as following: 94 °C for 2 min, 35 circles of 95 °C for 5 s, 55 °C for 30 s, 72 °C for 30 s and 68 °C for 5 min. Each sample was performed with three biological replicates. The results were calculated by 2^−ΔΔCt^ algorithm.

### Enzyme-linked immunosorbent assay

To monitor the accumulation of SMV-SC15 in transgenic plants, Enzyme-linked Immunosorbent assay (ELISA) was applied for T_2_ seedlings. The kits (complete with anti-SMV antibodies) were bought from ACD Inc., Fayetteville, AR, USA (cat #V094-R1). The leaves of 10 inoculated T_2_ seedlings (single copy in southern blot) were collected in 15 days past inoculation (dpi) and 30 dpi to detect SMV-SC15. Non-inoculated Tianlong No.1 was negative control and the leaves with SMV-SC15 symptoms were positive control. The optical density of samples at 405 nm wavelength was read in ELISA. The samples with values two times more than those of negative control were designated as susceptible to SMV.

### Statistical analysis

To reveal the heredity pattern of T_1_ seedlings, Chi square (*χ*^2^) test was carried out using SAS program (SAS Institute v. 9.2). Only the lines with more than 5 progenies were selected. The segregation ratios were calculated as positive progenies divided by negative progenies with greatest *P* value to test the goodness of fits of 3:1 (single functional loci), 15:1 (two standalone loci) and 1:1 (abnormal heredity pattern). The segregation ratios did not match with the above-mentioned ratios were designated as other heredity pattern. For seed mottling assay, twenty transgenic plants and fifteen non-transgenic plants were harvested separately and the mottling seeds of each plants were monitored to generate the average seed coat mottling rate. *T* test was used and p < 0.01 was significant differences.

## Results

### Screening of T_0_ positive transgenic soybean seedlings

**Fig. 1:**
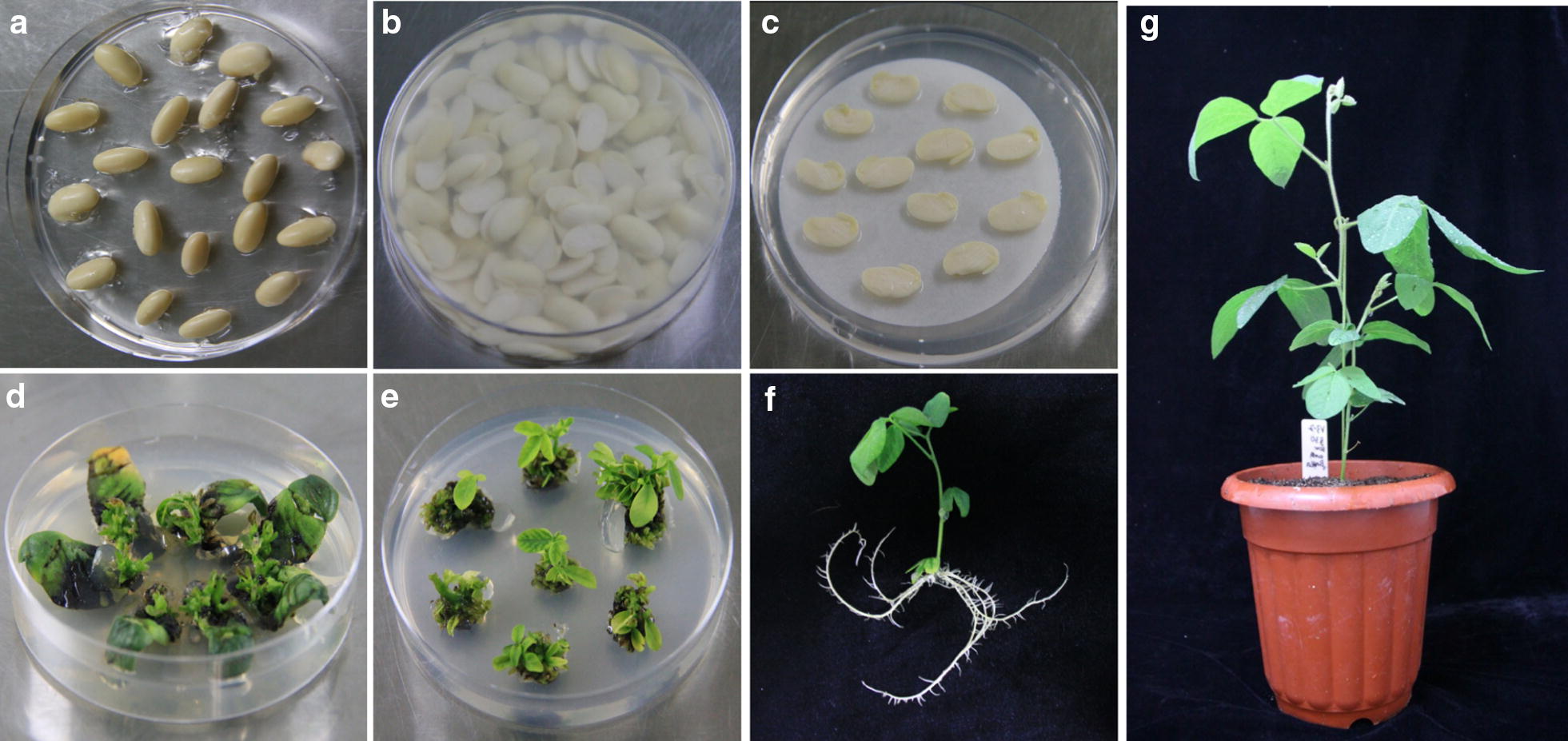
Process of *Agrobacterium*-mediated soybean cotyledonary-node transformation. **a** Seeds germination. **b** Incubated in LCCM. **c** Co-cultivation. **d** Shoot induction. **e** Shoot elongation. **f** Rooting. **g** Seedling survival

The steps of soybean transformation were displayed in Fig. 1. The buds of germinated seeds were cut out and used for transformation (Fig. 1a, b). The cotyledons were laid on co-cultivation medium (CCM) with filter paper (Fig. 1c) and incubated for 5 d in dark. Then, the explants were moved to shoot induction medium (SIM) including 5 mg/L phosphinothricin (PPT) and clustered shoots could be observed around 4 weeks after transplanting (Fig. 1d). The clustered shoots were resected and the explants were moved onto shoot elongation medium (SEM), which were sub-cultured every 2 weeks (Fig. 1e). The shoots which elongated more than 4 cm were clipped and inserted into rooting medium (RM). Roots were monitored after 10 days of elongation (Fig. 1f). Then, the seedlings were grafted to nutrient soil and used for positive seedling detection (Fig. 1g).

**Fig. 2:**
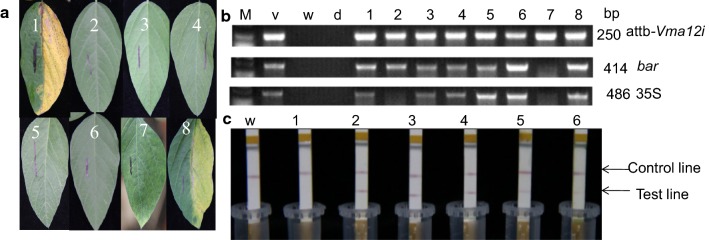
Identification of T_0_ transgenic soybean plants. **a** Identification of T_0_ transgenic soybean plants by herbicide painting, 1: wild type Tianlong No.1; 2–7: herbicide resistant plants; 8: herbicide nonresistant plant. **b** Identification of T_0_ transgenic soybean plants by PCR, M: Marker D2000; v: positive control of plasmid; w: negative control of wild-type Tianlong No.1; d: blank control of ddH_2_O; 1, 3–6 and 8: positive transgenic plants; 2 and 7: negative transgenic plant. **c** Identification of T_0_ transgenic soybean plants by QuickStix Kit for Liberty Link (bar), w: negative control of wild-type Tianlong No.1; 1: negative transgenic plants; 2–6: positive transgenic plants

The results of transformation and positive seedling screening were described in Table 1, and a total of 33 positive T_0_ transgenic seedlings were obtained. The PCR products were detected by Agarose Gel Electrophoresis. The samples that could be amplified by three pairs of primers were considered as positive seedlings, indicating successful integration of the vector into the soybean genome (Fig. 2b). In leaf-painting assay, 200 mg/L PPT was applied on half of the leaf (with mark line). The positive leaves displayed no difference with the other half, however wilting appeared on the leaves of non-transgenic negative control and false positive seedlings (Fig. 2a). The result of LibertyLink^®^ strip assay showed that both control line and positive line were observed on the strips of positive sample (Fig. 2c), indicating *bar* resistant gene was expressed.

**Table 1:** Results from screening positive plants in T_0_ and T_1_*GmVma12* transgenic generations

Serial number	Explant number	Rooting plants	Seedling number	Number of positive T_0_ plants^a^	Number of T_1_ plants	Number of positive T_1_ plants^a^
1	103	13	8	8	37	32
2	135	27	9	8	64	58
3	256	9	4	3	24	21
4	210	8	6	4	49	27
5	185	9	5	5	18	16
6	238	10	5	5	17	6
Total	1127	76	37	33	209	160

^a^All of the positive plants were confirmed by leaf-painting assay, PCR verification and LibertyLink^®^ strip detection

**Fig. 3:**
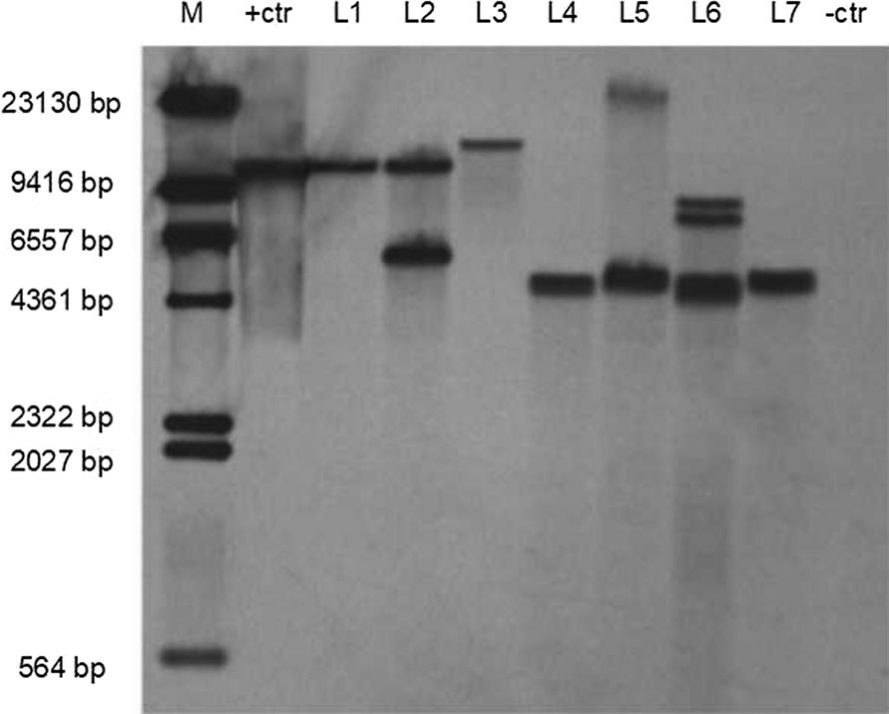
Southern blot analysis of T_1_ plants. M: DNA molecular markers; +ctr: vector was used as the positive control; L1–L7 indicate genomic DNA of T_1_ plants from T_0_ line No. 3, No. 5, No. 7, No. 12, No. 14, No. 16, No. 17; -ctr: genomic DNA sampled from non-transformed soybean plants was used as the negative control

To analyze the number of insertions of *GmVma12i*, healthy T_1_ plants from each of the seven transgenic T_0_ lines were selected randomly for southern blot analysis (Fig. 3). The result showed that all of the bands had the expected size of bigger than 3.5 Kb. The L1 and L3 contained single copy insertion, L2, L4, L5, L7 had two copies insertion and L6 exhibited quadruple insertion. Non-transformed plant (-ctr) did not contain insertion.

### Segregation analysis of heredity pattern in T_1_ generation

In the *agrobacterium*-mediated soybean cotyledon node transformation, a total of 1127 explants were infected and 37 seedling plants were obtained. Among them, 33 plants were detected as positive T_0_ transgenic plants. Subsequently, 209 T_1_ generation plants were obtained and 160 positive transgenic plants were identified (Table 1).

Subsequently 183 T_1_ plants from 12 T_0_ lines were analyzed by Chi square (*χ*^2^) test (Table 2). Among those lines, six segregated in the 3:1 ratio and three segregated in 15:1 ratio, which fit the Mendelian genetic law for one or two pairs of alleles. Additionally, five lines segregated in an abnormal way (1:1 or other, data not shown). Based on the insertion number and segregation ratio, the lines No.3 and No.7 were chosen for further study.

**Table 2:** Segregation analysis of 209 T_1_*GmVma12* transgenic plants progenies

T_0_ No.	T_1_ seg. ratio^a^	Best fit	(χ2)	P-value
12	4:4	3:1	0.2667	0.6056
21	4:5	3:1	0.7073	0.4003
26	6:4	3:1	0.0570	0.8113
3	27:7	3:1	0.0209	0.8851
17	11:2	3:1	0.0149	0.9028
20	21:6	3:1	0.0064	0.9362
7	6:2	3:1	0.3333	0.5637
23	9:2	3:1	0.0168	0.8969
21	9:2	3:1	0.0168	0.8969
24	27:2	15:1	0.1853	0.6668
22	7:0	15:1	0.7465	0.3876
28	15:1	15:1	0.5333	0.4652

^a^T_1_ seg. ratio indicates T_1_ segregation ratio (positive: negative)

### Evaluation of resistance to SMV in T_1_ and T_2_ plants

**Fig. 4:**
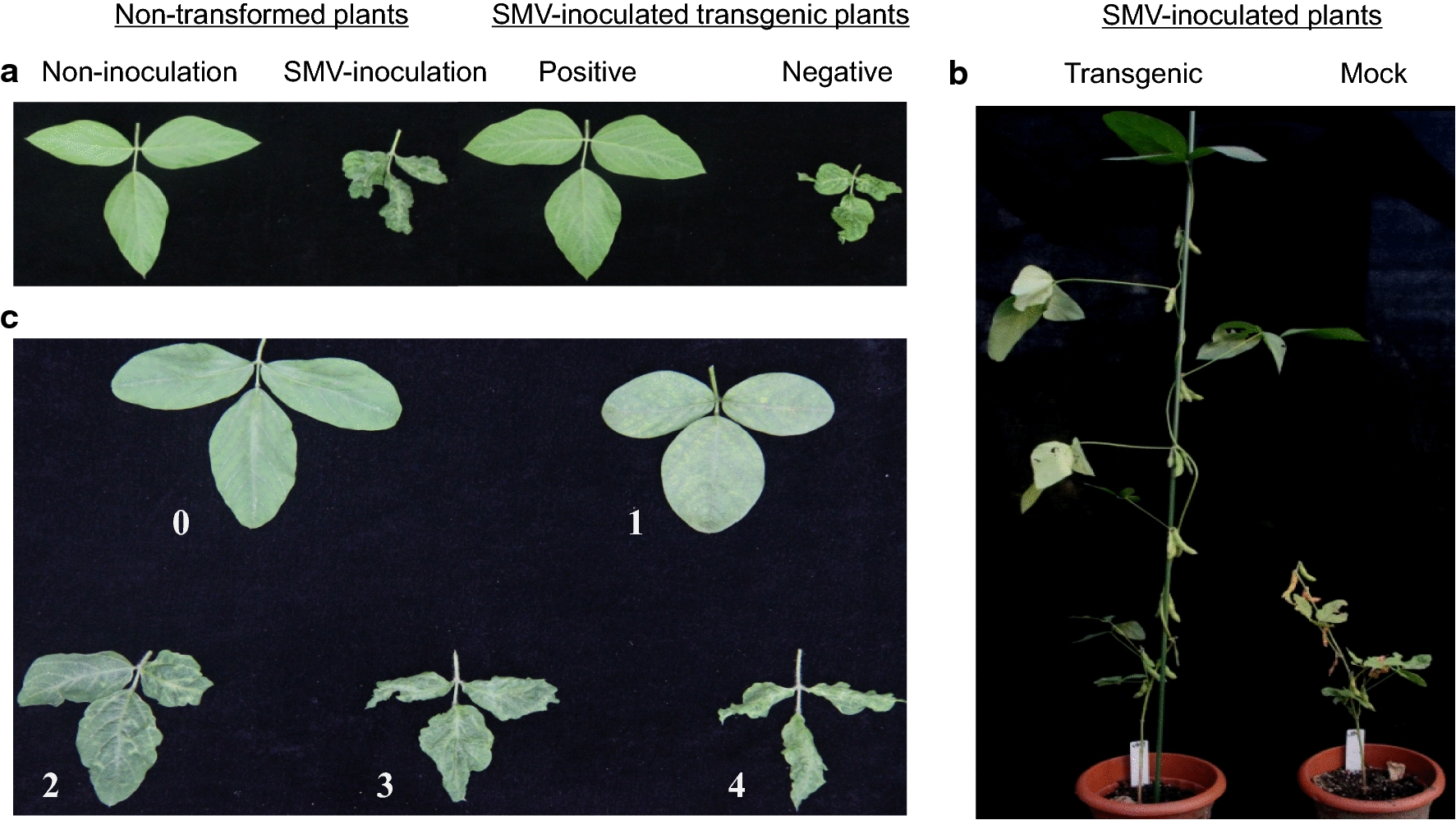
Symptoms of T_1_ plants after inoculating SMV-SC15 and disease rating classification. **a** Symptoms on the V4 leaves. **b** Response of T_1_ transformed plants and non-transgenic plants in 8 weeks after inoculation with SMV. **c** SMV disease rating was classified to five (0–4) levels

To investigate the impact of knocking down *GmVma12* on viral resistance, T_1_ and T_2_ plants were inoculated with SMV-SC15 on the true leaves, and the symptom of V_1_–V_4_ were inspected successively and classified at 7 dpi. In T_1_ generation, a total of 160 positive plants were examined. As shown in Table 3, almost a quarter of plants (41 plants) were identified as high resistance (HR). In T_2_ generation, 38 plants from two lines were examined and 23 plants were HR (60.54%). Meanwhile, plants showing mild resistance (MR) and delayed resistance (DR) were also found in transgenic population. MR was the main phenotype in T_1_ generation, accounted up to 49.37%. To quantify the resistance of transgenic plants during reproductive growth stage, the disease severity of top three trefoil were classified into five grades were classified (0–4, Fig. 4c). The results showed that the average disease ratings of T_1_ and T_2_ plants were 1.12 and 1.08 (mild mosaic), respectively, which were significantly lower than the ratings of non-transformed plants (3.07 and 3.20, crinkle mosaic).

**Table 3:** Response types of T1 and T2 positive plants at V1–V4 stage

Generation No.	Response types				Total
	HR^a^	MR^b^	DR^c^	S^d^	
T1	25.63% (41)	49.37% (79)	8.75% (14)	16.25% (26)	160
T2	60.54% (23)	23.68% (9)	7.89% (3)	7.89% (3)	38

^a^*HR* high resistance, indicating that no any visible symptoms appeared on the observed leaves

^b^*MR* delayed resistance, indicating that symptoms could be observed at V_1_ and V_2_ stage, but not at V_3_ and V_4_ stage

^c^*DR* mild resistance, indicating that the symptoms appearing on the observed leaves were lighter than the symptoms of susceptible plants

^d^*S* susceptible, indicating the symptoms were obvious and severe, which were similar to negative control

The phenotype of non-transformed plants and transgenic plants were observed. Knocking down the *GmVma12* did not change the morphology of soybean at both true leaf period stage and pod setting stage (Additional file 1: Fig. S2). The leaves of SMV-inoculated transgenic plants with high resistance was as healthy as non-inoculated leaves of wild type, but the negative transgenic plants were as susceptible as SMV inoculated wild type plants (Fig. 4a). SMV had a severe impact on the growth of soybeans. As shown in Fig. 4b, non-transformed susceptible plants and negative transgenic plant were dwarf. However, SMV inoculated positive transgenic plant was as healthy as uninoculated non-transformed plants.

**Fig. 5:**
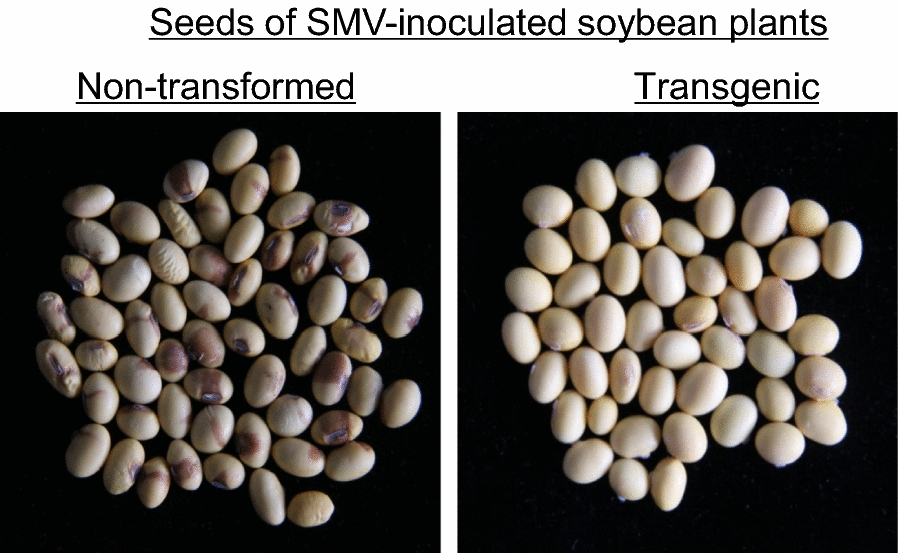
Investigation of seed coat mottling from transgenic plants and non-transgenic plants

SMV infection also forms mottle on seed coat (Lim et al. 2007; Senda et al. 2004). Therefore, we randomly investigated the seeds of 20 positive T_2_ plants and 15 SMV-inoculated non-transformed plants to illustrate the resistance to SMV (Fig. 5). The statistical results showed that the average seed coat mottling rate of positive T_2_ plants was 2.82%, much lower than that of negative control (81.39%). The average seed coat mottling rate further demonstrated that the resistance to SMV in Tianlong No.1 transgenic plants was significantly improved.

### Resistance to SMV was induced by knock-down *GmVma12* in soybean

**Fig. 6:**
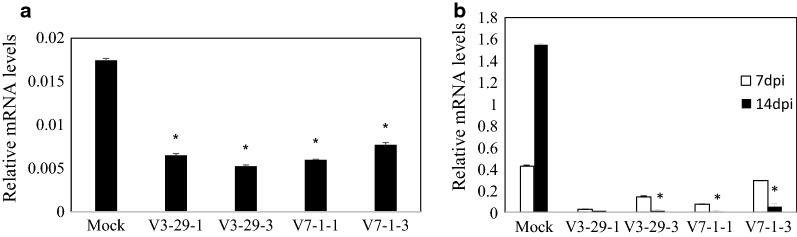
Molecular detection of T_2_ plants. **a***GmVma12* expression level in T_2_ RNAi silenced transgenic plants by qRT-PCR. **b** SMV accumulation in SMV-infected T_2_ transgenic plants by qRT-PCR. Y-axes indicate the transcript levels of *GmVma12* (7 dpi) and SMV (7 dpi and 14dpi) of SMV-infected plants. X-axes indicate T_2_ transgenic plants and non-transformed (Mock) plants. Data are expressed as the means of three biological replicates with error bars indicating the SD (n = 3). Asterisks denote significant difference from mock, as determined by the *t*-test, *p *< 0.001. Each result is representative of three biological repeats

The expression of *GmVma12* and SMV-CP in T_2_ population were tested by qRT-PCR. The results showed that the expression of *GmVma12* in T_2_ plants decreased up to 70% (V3-29-3) as compared with negative control at 7 dpi, indicating successful knocking down of *GmVma12* at the transcriptional level (Fig. 6a). While the other isoforms was also knocked down (Additional file 1: Fig. S3). Meanwhile, the expression of SMV-*CP* in transgenic plants were also lower than that in non-transformed plants at 7 dpi and 14 dpi, indicating that SMV replication was inhibited in transgenic plants. Furthermore, the accumulation of SMV in transgenic plants reduced more at 14 dpi than that at 7 dpi. (Fig. 6b).

Enzyme-linked Immunosorbent assay was performed for plants at 15 dpi and 30 dpi, respectively, to test the accumulation of SMV. As showed in Table 4, all the OD_405nm_ values of the analyzed T_2_ transgenic plants after 15 days SMV inoculation were less than those of negative control, meaning that SMV was not detected in transgenic plants. In contrast, the OD_405nm_ values of positive control were 10 times more than those of negative control. The same results were also observed at 30 dpi (Additional file 1: Table S3). In summary, the results of qRT-PCR and ELISA analyses demonstrated that knocking down the expression of *GmVma12* could be responsible for the induction SMV resistance in soybean.

**Table 4:** DAS-ELISA analyses of T_2_ plants based on the optical density value (OD_405nm_)

No.	P^a^(OD_405_nm*)*	N^b^(OD_405_nm)	P/N
1	0.10	0.11	0.91 (–)
2	0.07	0.11	0.63 (–)
3	0.02	0.11	0.18 (–)
4	0.13	0.11	1.18 (–)
5	0.05	0.11	0.45 (–)
6	0.07	0.11	0.63 (–)
7	0.09	0.11	0.82 (–)
8	0.01	0.11	0.09 (–)
9	0.01	0.11	0.09 (–)
10	0.02	0.11	0.18 (–)
WT	1.17	0.11	10.64 (+)

(+) positive for SMV, (−) negative for SMV

^a^The OD405nm values of SMV-inoculated T2 plants

^b^The OD405nm values of Non-inoculated wild type (WT) plants

## Discussion

The GmVma12 was knocked down in soybean cultivar Tianlong No. 1 by *agrobacterium*-mediated transformation. By PCR, PPT painting and LibertyLink^®^ strip screening for the positive transgenic plants and SMV resistance evaluation, L3 and L7 were selected as HR genotype plants which will be used for homozygous identification. Since the *agrobacterium*-mediated transformation method was applied to obtain the first transgenic soybean in 1988 for the first time, this technology has been widely used in soybean transgenic research (Hinchee et al. 1988). Olhoft used germinated 5–7 d soybean cotyledonary nodes as explants, added thiol compounds such as l-Cysteine, copper and iron chelators to medium (Olhoft et al. 2001, 2001) and screened by hygromycin B (Olhoft et al. 2003), which increased the transformation efficiency up to 16.4%. In our study, the rate of transformation was around 3%, lower than the other reported transformation assays. We found that the rooting seedlings were sensitive to changes of environment, since half of them died after transplantation from the culture chamber to the pots. Therefore, we would focus on cultivating stronger seedlings in RM, improving the handling steps of acclimation and transplanting and optimizing the conditions of culture room and artificial climate chamber to increase transformation rate.

Existing positive detection methods rely on molecular features such as mRNA and protein expression, and phenotypic observations, but the results of these detection methods were sometimes inconsistent. In the present study, some positive plants identified by herbicide were negative in LibertyLink^®^ strip test. Olhoft found a chimeric phenomenon in the progeny of transgenic soybeans (Olhoft et al. 2003). The chimera was produced because only some cells of the shoots contained expression vector; hence, only part of plant was positive. In screening positive transgenic seedlings assay, leaves were selected randomly, so it was speculated that the chimeric plants caused inconsistent result. Therefore, the detection of the reporter gene should be repeated and additional methods such as Southern Blot or Western Blot should be used to analyze the integration or translation of the transgene.

During the statistical analysis of the transgenic T_1_ seedlings, we found that the heredity pattern of some lines were not based on the genetic law of one or two pairs of alleles of Mendelian segregation patterns, and some plants did not even transfer the T-DNA to offspring. This observation might be due to chimerism discussed above, gene silencing, non-transformed bud escaping the screen, unstable T-DNA integration site and limited number of progenies (Olhoft et al. 2003; Rong et al. 1996). The segregation ratio of T_0_ line No. 3 and line No. 7 were both 3:1 (Table 2). This segregation ratio matched with the single copy insertion results of Southern Blot (Fig. 6), which indicated that the progenies of these two lines could generate stable and inheritable transgenic lines.

RNA interference is a natural defense response of plants to viral infections (Tenllado et al. 2003). At present, RNAi has mainly been applied on the coat protein of virus, movement protein, the replicase, and the dominant or recessive disease resistance genes of host plants. Transgenic plants have great differences in virus resistance, and are mainly classified into high resistance, low resistance, susceptible and recovery. The coat protein of *Tobacco etch virus* (TEV) was transformed into tobacco (Goodwin et al. 1996). After inoculation with TEV, the virus was found in the plants in a short time. However, with the passage of time, the accumulation of viruses in new leaves gradually decreased. This phenomenon is called “recovery”, which was speculated related to transgene copy number(Goodwin et al. 1996). The qRT-PCR and DAS-ELISA assay showed that the silencing efficiency of the endogenous genes was not 100%, which indicated that their expression was inhibited to some extent (Fig. 6). In our experiment, the similar penotype was also found and named delay resistance(DR). The silencing efficiency of *GmVma12i* and accumulation of SMV in DR plants were also detected (no-show data), which was lower than HR plants. We assumed that the expression of RNAi construction and the efficiency of siRNAs were also important for the resistance phenotype. Moreover, the strength of delay resistance could also be parallel with the SMV accumulation. The severe symptom of SMV in early growth stage might attenuate DR (Furutani et al. 2007).

The vacuolar-type ATPase (VATP) is prevalent in the endometrium of eukaryotic and animal cells. This enzyme functions as a proton pump and plays an important role in the membrane system, including membrane trafficking and intracellular pH regulation (Beyenbach et al. 2006; Forgac et al. 2007). VATP contains multiple subunits that belong to the heterologous polyproteinases. In intracellular vacuolar transport, which is mediated by organelles such as the endoplasmic reticulum, Golgi apparatus, and lysosomes, VATP transports newly generated secretory proteins and receptor proteins to the plasma membrane (Bonifacino et al. 2004). Meanwhile, VATP also plays a key role in other intracellular transport pathways, such as coordinating the interaction between intracellular organ receptors and extracellular donor organs, organ fusion, budding, and secretion (Baars et al. 2007; Marshansky et al. 2008; 2014). In the Mannose-6-phosphate (M6P) pathway, VATP is involved in the synthesis and transport of hydrolases, and regulates the transport from the Golgi apparatus or the endoplasmic reticulum to lysosomes (Maxfield et al. 2004; Coutinho et al. 2012). Until now, there are no reports about the function of Vma12 related to disease resistance. It was obvious that Tianlong No.1 gained the resistance to SMV by knocking down *Gmvma12*, which implicates *GmVma12* plays an important role in the invasion of SMV as a host factor.

In the future study, the stable homozygous line with stable resistance to SMV could be screened and used to study the role of *GmVma12* during SMV infection, replication and transport. Moreover, it will be interesting to investigate the variation of ultrastructure in different stages after SMV inoculation, analyze the changes of expression of pathogenesis-related proteins and search for related pathways to clarify how *GmVma12* functions in the process of SMV invasion.

## Supplementary information

**Additional file 1: Fig. S1.** Schematic diagram of T-DNA region of recombinant plasmid pB7GWIWG2(II)-*GmVma12i,***Fig. S2.** The morphology of **Fig. S3.** The transcript levels of two isforms of *GmVma12* by qRT-PCR in T_1_ RNAi silenced transgenic plants qRT-PCR. X-axes indicate T_1_ transgenic plants and non-transformed (Mock) plants. Data are expressed as the means of three biological replicates with error bars indicating the SD (n = 3). Asterisks denote significant difference from mock, as determined by the t- test, *p* < 0.001. Each result is representative of three biological repeats. wild type and GmVma12 transgenic plants at (a) true leaf period stage and (b) pod setting stage. **Table S1.** Primer sequence. **Table S2.** Components in medium used for soybean transformation. **Table S3.** DAS-ELISA analyses of T_2_ plants at 30 dpi of SMV infection

## Data Availability

All data generated or analyzed during this study are included in the figures and tables. Any material used in this study is available for research purposes upon request.
