# Supplementary material for: Transgenic plant generated by RNAi-mediated knocking down of soybean Vma12 and soybean mosaic virus resistance evaluation
Source: AMB Express. 2020 Apr 6;10:62. doi: 10.1186/s13568-020-00997-6 (PMC7136382; doi:10.1186/s13568-020-00997-6)
Supplement: Supplementary file 1 — Additional file 1: Fig. S1. Schematic diagram of T-DNA region of recombinant plasmid pB7GWIWG2(II)-GmVma12i,Fig. S2. The morphology of Fig. S3. The transcript levels of two isforms of GmVma12 by qRT-PCR in T1 RNAi silenced transgenic plants qRT-PCR. X-axes indicate T1 transgenic plants and non-transformed (Mock) plants. Data are expressed as the means of three biological replicates with error bars indicating the SD (n = 3). Asterisks denote significant difference from mock, as determined by the t- test, p < 0.001. Each result is representative of three biological repeats. wild type and GmVma12 transgenic plants at (a) true leaf period stage and (b) pod setting stage. Table S1. Primer sequence. Table S2. Components in medium used for soybean transformation. Table S3. DAS-ELISA analyses of T2 plants at 30 dpi of SMV infection [file 13568_2020_997_MOESM1_ESM.docx]

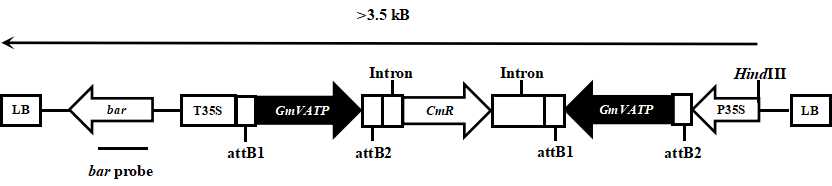


Fig. S1. Schematic diagram of T-DNA region of recombinant plasmid pB7GWIWG2(II)-*GmVma12i*


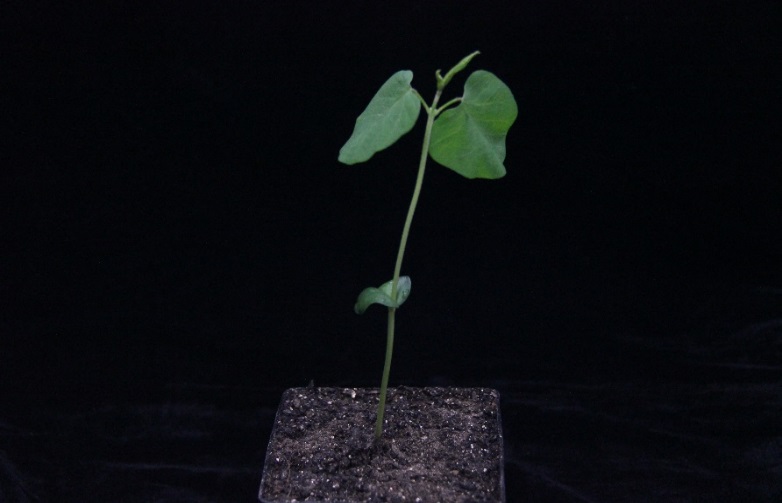

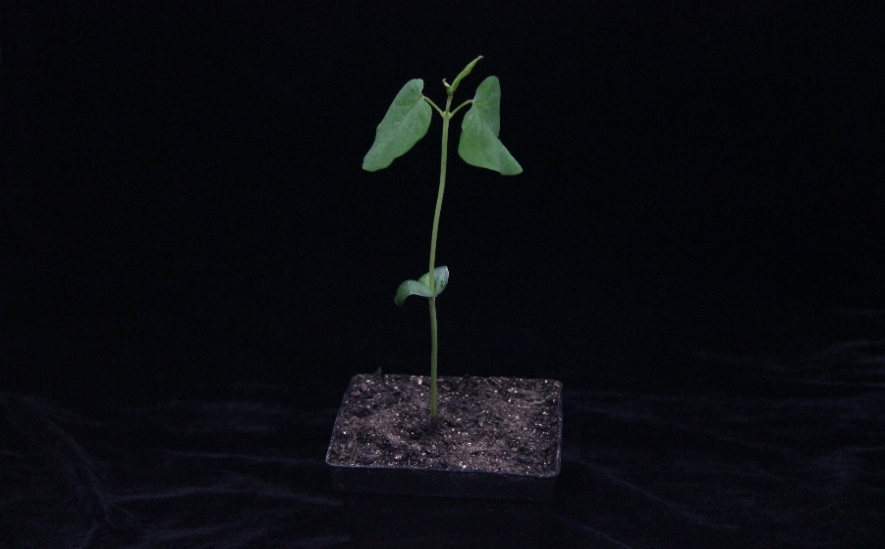


WT Transgenic

a

b


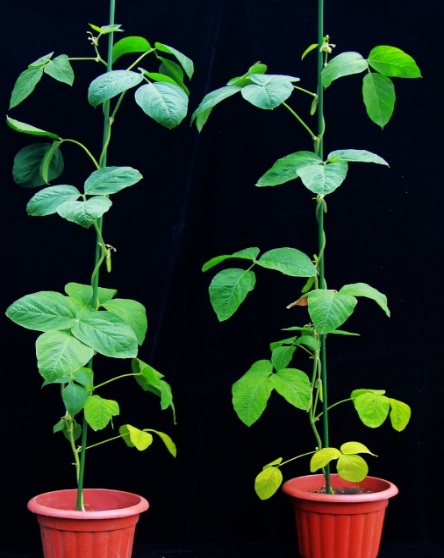


**Fig. S2 The** morphology of wild type and GmVma12 transgenic plants at (a) true leaf period stage and (b) pod setting stage.

**Fig. S3** The transcript levels of two isforms of *GmVma12* by qRT-PCR in T_1_ RNAi silenced transgenic plants qRT-PCR. X-axes indicate T_1_ transgenic plants and non-transformed (Mock) plants. Data are expressed as the means of three biological replicates with error bars indicating the SD (n = 3). Asterisks denote signiﬁcant difference from mock, as determined by the *t*-test, *p*<0.001. Each result is representative of three biological repeats.

Table S1. Primer sequence

| Gene name | Primer sequence （5′→3′） |
| --- | --- |
| GmVma12i F | GGGGACAAGTTTGTACAAAAAAGCAGGCTTCGGTCGGGTTAGTGATATCCA |
| GmVma12i R | GGGGACCACTTTGTACAAGAAAGCTGGGTCAGTCGGGTCAAGTCGGGTCT |
| 35S F | GCTCAACACATGAGCGAAAC |
| 35S R | GACGCACAATCCCACTATCC |
| Bar F | CGAGACAAGCACGGTCAACTT |
| Bar R | AAACCCACGTCATGCCAGTTC |
| probe F | GAGAATTAAGGGAGTCACGTTATG |
| probe R | CGTTGCGTGCCTTCCAG |
| Tubulin F | GGAGTTCACAGAGGCA GAG |
| Tubulin R | CACTTACGCATCACATAGCA |

Table S2. Components in medium used for soybean transformation

| Component | GM | LCCM | CCM | LSIM | SIM | SEM | RM |
| --- | --- | --- | --- | --- | --- | --- | --- |
| B5 salts | 3.21 g/L | 0.321 g/L | 0.321 g/L | 3.21 g/L | 3.21 g/L | – | – |
| MS salts | – | – | – | – | – | 4.43 g/L | 2.22 g/L |
| MES | 3 mM | 20 mM | 20 mM | 3 mM | 3 mM | 3 mM | 3 mM |
| Sucrose | 2% | 3% | 3% | 3% | 3% | 3% | 2% |
| Noble agar | – | – | 0.5% | – | – | – | – |
| Phytagel | 0.3% | – | – | – | – | – | 0.3% |
| Agar | – | – | – | – | 0.8% | 0.8% | – |
| PH | 5.8 | 5.4 | 5.4 | 5.7 | 5.6 | 5.6 | 5.6 |
| BAP | – | 1.67 mg/L | 1.67 mg/L | 1.67 mg/L | 1.67 mg/L | – | – |
| GA_3_ | – | 0.25 mg/L | 0.25 mg/L | – | – | 0.5 mg/L | – |
| As | – | 0.2 mM | 0.2 mM | – | – | – | – |
| L-Cys | – | 3.3 mM | 3.3 mM | – | – | – | – |
| DTT | – | 1.0 mM | 1 mM | – | – | – | – |
| Glufosinate | – | – | – | – | 5 mg/L | 5 mg/L | – |
| Timentin | – | – | – | 250 mg/L | 250 mg/L | 250 mg/L | – |
| Cefotaxime | – | – | – | 100 mg/L | 100 mg/L | 100 mg/L | 100 mg/L |
| Asparagine | – | – | – | – | – | 50 mg/L | – |
| Glutamine | – | – | – | – | – | 50 mg/L | – |
| IAA | – | – | – | – | – | 0.1 mg/L | – |
| Zeatin-riboside | – | – | – | – | – | 1 mg/L | – |
| IBA | – | – | – | – | – | – | 0.1 mg/L |

“–” means no added

Table S3. DAS-ELISA analyses of T_2_ plants at 30 dpi of SMV infection

| No. | *P^a^(OD_405_nm)* | *N^b^(OD_405nm_)* | *P/N* |
| --- | --- | --- | --- |
| 1 | 0.11 | 0.19 | 0.58(-) |
| 2 | 0.01 | 0.19 | 0.05(-) |
| 3 | 0.10 | 0.19 | 0.53(-) |
| 4 | 0.10 | 0.19 | 0.53(-) |
| 5 | 0.13 | 0.19 | 0.68(-) |
| 6 | 0.01 | 0.19 | 0.05(-) |
| 7 | 0.01 | 0.19 | 0.05(-) |
| 8 | 0.11 | 0.19 | 0.58(-) |
| 9 | 0.12 | 0.19 | 0.63(-) |
| 10 | 0.15 | 0.19 | 0.79(-) |
| WT | 1.53 | 0.19 | 8.05(+) |

(+) positive for SMV, (−) negative for SMV

a: the OD405nm values of SMV-inoculated T2 plants

b: the OD405nm values of Non-inoculated wild type (WT) plants
